# Cerebral near-infrared spectroscopy monitoring versus treatment as usual for extremely preterm infants: a protocol for the SafeBoosC randomised clinical phase III trial

**DOI:** 10.1186/s13063-019-3955-6

**Published:** 2019-12-30

**Authors:** Mathias Lühr Hansen, Adelina Pellicer, Christian Gluud, Eugene Dempsey, Jonathan Mintzer, Simon Hyttel-Sørensen, Anne Marie Heuchan, Cornelia Hagmann, Ebru Ergenekon, Gabriel Dimitriou, Gerhard Pichler, Gunnar Naulaers, Guoqiang Cheng, Hercilia Guimarães, Jakub Tkaczyk, Karen B. Kreutzer, Monica Fumagalli, Olivier Claris, Petra Lemmers, Siv Fredly, Tomasz Szczapa, Topun Austin, Janus Christian Jakobsen, Gorm Greisen

**Affiliations:** 1 Department of Neonatology, Rigshospitalet, Blegdamsvej 9, 2100 Copenhagen, Denmark; 2 Department of Neonatology, La Paz University Hospital, Paseo De La Castellana 261, 28046 Madrid, Spain; 3 Copenhagen Trial Unit, Rigshospitalet, Blegdamsvej 9, 2100 Copenhagen, Denmark; 4 Infant Centre and Department of Paediatrics and Child Health, University College Cork, College Road, Cork, Ireland; 5 Division of Newborn Medicine, Hackensack Meridian Health – Mountainside Medical Center, 1 Bay Ave, Montclair, NJ USA; 6 Department of Intensive Care, Rigshospitalet, Blegdamsvej 9, 2100 Copenhagen, Denmark; 7 Department of Neonatology, Royal Hospital for Children, 1345 Govan Rd, Glasgow, G51 4TF UK; 8 Department of Neonatology, Children’s University Hospital of Zürich, Steinweisstrasse 75, 8037 Zurich, Switzerland; 9 Department of Neonatology, Gazi University Hospital, Emniyet Mahallesi, Gazeteci Yazar Muammer Yaşar Bostancı Sokak, 06560 Yenimahalle/Ankara, Turkey; 10 NICU, Department of Pediatrics, University General Hospital of Patras, 265 04 Patras, Greece; 11 Department of Pediatrics, Medical University of Graz, Auenbruggerplatz 30, Graz, Austria; 12 Department of Neonatology, University Hospital Leuven, Herestraat 49 Leuven, Belgium; 13 Department of Neonatology, Children’s Hospital of Fudan University, 399 Wanyuan Rd, Minhang Qu, Shanghai Shi, China; 14 Department of Neonatology, Centro hospitalar Universitário de São João, Alameda Prof. Hernâni Monteiro, 4200-319 Porto, Portugal; 15 Department of Neonatology, University Hospital Motol, V Uvalu 84, 150 06 Prague 5, Czech Republic; 16 Department of Neonatology, University Children’s Hospital Tuebingen, Hoppe-Seyler-Straße 1, 72076 Tuebingen, Germany; 17 Fondazione IRCCS Ca’ Granda Ospedale Maggiore Policlinico Milan, Via della Commenda 12, IT- 20122 Milan, Italy; 18 Department of Clinical Sciences and Community Health, University of Milan, Via Francesco Sforza 35, 20122 Milan, Italy; 19 Department of Neonatology, Hospices Civil De Lyon, 3 Quai des Célestins, 69002 Lyon, France; 20 Department of Neonatology, Wilhelmina Children’s Hospital, Lundlaan 6, 3584 EA Utrecht, Netherlands; 21 Department of Neonatology, Oslo University Hospital, Kirkeveien, 166 0450 Oslo, Norway; 22 Department of Neonatology, Poznan University of Medical Sciences, Polna 33, 60-535 Poznań, Poland; 23 Neonatal Intensive Care Unit, Cambridge University Hospitals NHS Foundation Trust, Hills Road, Cambridge, CB2 0SW UK; 24 Department of Cardiology, Holbæk Hospital, Smedelundsgade 60, 4300 Holbæk, Denmark; 25 Department of Regional Health Research, The Faculty of Health Sciences, University of Southern Denmark, Odense, Denmark

**Keywords:** Randomised clinical trial, Preterm, Near infrared spectroscopy, Protocol

## Abstract

**Background:**

Cerebral oxygenation monitoring may reduce the risk of death and neurologic complications in extremely preterm infants, but no such effects have yet been demonstrated in preterm infants in sufficiently powered randomised clinical trials. The objective of the SafeBoosC III trial is to investigate the benefits and harms of treatment based on near-infrared spectroscopy (NIRS) monitoring compared with treatment as usual for extremely preterm infants.

**Methods/design:**

SafeBoosC III is an investigator-initiated, multinational, randomised, pragmatic phase III clinical trial. Inclusion criteria will be infants born below 28 weeks postmenstrual age and parental informed consent (unless the site is using ‘opt-out’ or deferred consent). Exclusion criteria will be no parental informed consent (or if ‘opt-out’ is used, lack of a record that clinical staff have explained the trial and the ‘opt-out’ consent process to parents and/or a record of the parents’ decision to opt-out in the infant’s clinical file); decision not to provide full life support; and no possibility to initiate cerebral NIRS oximetry within 6 h after birth. Participants will be randomised 1:1 into either the experimental or control group. Participants in the experimental group will be monitored during the first 72 h of life with a cerebral NIRS oximeter. Cerebral hypoxia will be treated according to an evidence-based treatment guideline. Participants in the control group will not undergo cerebral oxygenation monitoring and will receive treatment as usual. Each participant will be followed up at 36 weeks postmenstrual age. The primary outcome will be a composite of either death or severe brain injury detected on any of the serial cranial ultrasound scans that are routinely performed in these infants up to 36 weeks postmenstrual age. Severe brain injury will be assessed by a person blinded to group allocation. To detect a 22% relative risk difference between the experimental and control group, we intend to randomise a cohort of 1600 infants.

**Discussion:**

Treatment guided by cerebral NIRS oximetry has the potential to decrease the risk of death or survival with severe brain injury in preterm infants. There is an urgent need to assess the clinical effects of NIRS monitoring among preterm neonates.

**Trial registration:**

ClinicalTrial.gov, NCT03770741. Registered 10 December 2018.

## Background

Every year, approximately 50,000 extremely preterm infants (< 28 weeks postmenstrual age) are born in countries where they routinely will be offered neonatal intensive care [1]. Extremely preterm birth carries a high risk of death or long-term cerebral impairment. With a current mortality of about 25% and a prevalence of psychomotor impairment in approximately 20% of survivors, more than 10,000 will die each year and a further 10,000 will suffer from cerebral palsy or moderate-to-severe cognitive impairment [2–4].

When an infant is born extremely preterm, all organs are immature and vulnerable [5, 6]. This is particularly relevant for the immature brain [7]. Cerebral autoregulation is limited and believed to be fragile in extremely preterm infants [4]. It is hypothesised that large fluctuations in cerebral blood flow may result in cerebral haemorrhage arising from immature blood vessels. These fluctuations in systemic blood flow are common during the transition from foetal to neonatal circulation during the first days of life, thus putting the immature brain in danger [8].

Neonatal brain injury may be diagnosed by cranial ultrasound [9]. The most severe injuries, including grade III or IV intraventricular haemorrhage and the non-haemorrhagic white matter injury cystic periventricular leukomalacia, entail a high probability of death or cerebral palsy [10, 11]. Several pre- and postnatal factors have been shown or are thought to be associated with cerebral injury, including ascending infections [12], insufficient nutrition early in life [13], insufficient blood pressure, cardiac dysfunction, and suboptimal mechanical ventilation [14–16].

Among extremely preterm infants during their first days of life, current practice standards involve multiple parallel interventions, including respiratory and haemodynamic support, intravenous fluids, antibiotics, nutrition, and monitoring of physiological parameters. Despite significant advances in the management of extremely preterm infants over the past three decades, many of these interventions are used with little evidence. Furthermore, an end-organ monitor with sufficient time resolution to guide evidence-based treatment is lacking. Near-infrared spectroscopy (NIRS) has the potential to function in this manner. Cerebral NIRS provides a real-time continuous estimate of the cerebral tissue oxygenation (rStO_2_), expressed as a percentage_._ The normal ranges of rStO_2_ in preterm infants have been determined and change somewhat with gestational age and postnatal age [17].

The evidence on the utility of NIRS monitoring in extremely preterm infants during the first days of life is sparse. Only one previous randomised clinical trial has assessed the effects of cerebral monitoring—the SafeBoosC phase II feasibility trial [18]. This trial showed that NIRS monitoring reduced the burden of cerebral hypoxia to less than half compared with treatment as usual and there were also non-significant trends towards reduced incidence of severe brain injury and reduced mortality in the NIRS group [18]. The clinical interventions used in the NIRS-open group included a significant number with likely beneficial effects on blood oxygen content and transport, blood pressure, cardiac output, and cerebral blood flow [19]. Despite these promising results, it is theoretically possible that NIRS monitoring may cause harm. This includes skin marks from the sensors, inappropriate modifications in cardio-respiratory support based on hypoxic values, and unnecessary infant disturbance due to manipulation of the forehead-based NIRS sensor. Furthermore, the SafeBoosc II trial showed a higher prevalence of bronchopulmonary dysplasia and retinopathy of prematurity in the experimental group. As NIRS devices and sensors are also costly and monitoring confers additional nursing tasks, it would be unfortunate to incorporate NIRS monitoring into standard practice without clear evidence of clinical benefit.

To evaluate the potential benefits and harms of NIRS monitoring, large-scale randomised clinical trials are urgently warranted. Since the intervention is complex—NIRS monitoring itself in addition to evidence-based modification of cardio-vascular support—a pragmatic design is preferable to ensure relevance for routine neonatal intensive care. International participation is additionally necessary to achieve adequate subject numbers and ideally promote generalisability of the results.

## Methods/design

This trial will be conducted in compliance with the guidelines of The Declaration of Helsinki in its latest form, the International Conference on Harmonization Good Clinical Practice guidelines [20], and applicable national regulations and directives. No clinical site will start randomisation before their eligibility has been confirmed and the protocol has been approved by the relevant ethics committee. Any amendments to the protocol will need approval by the Steering Committee and ethical review before being implemented. Written informed consent will be obtained by a qualified physician or nurse connected to the trial, prior to randomisation of any participant, unless the Neonatal Intensive Care Unit (NICU) uses deferred informed consent or prior assent as consent methods (see below). These consent procedures will be approved by local ethics committees or institutional review boards.

### Objective

The objective of this trial is to examine the benefits and harms of treatment based on NIRS monitoring compared with treatment as usual (standard monitoring and treatment) to reduce cerebral hypoxia during the first 72 h of life in extremely preterm infants. The hypothesis is that the application of treatment based on NIRS monitoring will decrease a composite outcome of severe brain injury or death at 36 weeks postmenstrual age.

### Roles and responsibilities for committees

SafeBoosC III is led by a Steering Committee comprising the coordinating investigator (GG), the national coordinators, and two representatives from the Copenhagen Trial Unit (CG and JCJ). Decisions will be made by a simple majority. The executive committee will be responsible for the day-to-day management and will comprise the coordinating investigator, the trial manager (MLH), co-investigators (AP, GD, JM, SHS), and the two representatives from the Copenhagen Trial Unit (CG and JCJ).

There will be one principal investigator in each department who will be responsible for obtaining ethical approval, organising local Good Clinical Practice monitoring, informing clinical staff members on the web-based training and certification program, recruitment of patients, and data entry into the patient report forms. The Copenhagen Trial Unit will be responsible for randomisation, development of the patient report forms, and central monitoring.

### Trial design

This is an investigator-initiated, multinational, randomised, pragmatic phase III clinical trial with a two-parallel group design that will enrol 1600 extremely preterm infants from 20 countries (Austria, Belgium, China, Czech Republic, Denmark, England, France, Germany, Greece, India, Ireland, Italy, Norway, Poland, Portugal, Switzerland, Scotland, Spain, Turkey, USA). A list of all study sites will be available at www.safeboosc.eu. It is an open label trial, but parts will be conducted blinded to the intervention (see the ‘Blinding’ section).

**Fig. 1:**
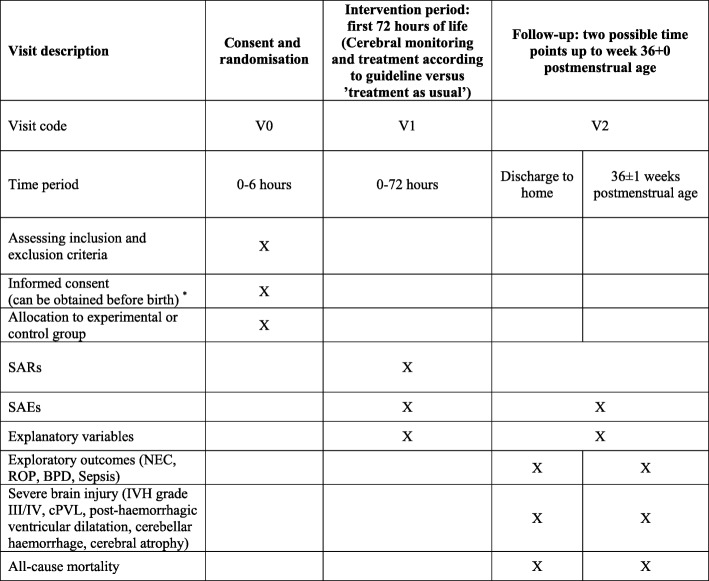
Schedule for enrolment, intervention and assessment, based on the SPIRIT 2013 guidance for protocols of clinical trials. *If approved by the local ethics committee, deferred informed consent or prior informed assent may be sought. Time to ask parents for deferred consent will be decided individually by clinical staff members

The trial has been designed according to the SPIRIT guidelines (Fig. 1 and Additional file 1) [21].

### Inclusion criteria

The inclusion criteria will be infants born before 28 weeks postmenstrual age and signed parental informed consent unless the NICU has chosen to use ‘opt-out’ or deferred consent as their consent method.

### Exclusion criteria

The exclusion criteria will be no signed parental informed consent (or if the ‘opt-out’ method is used, lack of a record that the clinical staff have explained the trial and the ‘opt-out’ consent process to parents and/or a record of the parents’ decision to opt-out in the infant’s clinical file); decision not to provide full life support; and no possibility to initiate cerebral NIRS monitoring within 6 hours after birth.

### Participation in other trials

Participants included in the SafeBoosC III trial can participate in any other study or intervention on the condition that: it does not allow clinical staff access to cerebral oximetry in the control group from inclusion in SafeBoosC III to the end of the intervention period 72 h after birth; and does not exclude a treatment that would be clearly indicated by the SafeBoosC III evidence-based treatment guideline during the intervention period. All partners are encouraged to design ancillary studies and draw on data collected by SafeBoosC III, if not compromising the blinding of assessors or the equipoise of the trial. Ancillary studies must seek approval by the SafeBoosC Steering Committee.

### Participant discontinuation and withdrawal

A participant’s parents are free to withdraw them from the SafeBoosC III trial at any time, and this will not have any consequences for the infant’s further treatment. Reasons for discontinuation, if provided by the parents, will be documented. When possible, the parents will be asked if they will allow their child’s data to be used in the analysis.

The attending clinician can withdraw the participant from the trial at any time in case there are safety concerns. Reasons for withdrawal will be documented. There are no pre-specified criteria for discontinuation of participants from the trial. Discontinuation of participants from the trial will not result in replacement with new participants.

### Recruitment

In this phase III trial, we have prolonged the enrolment period from 3 hours, as used in SafeBoosC II, to 6 hours after birth, although we recommend that monitoring is started as early as possible to help decision-making when cardio-respiratory support is established. This 6-hour window is similar to what is currently used for another neonatal intervention—therapeutic hypothermia for hypoxic-ischaemic encephalopathy after birth asphyxia [22]. We believe this will make the trial relevant in settings where antenatal transfer to a perinatal centre is used less often, and thereby increase recruitment feasibility without compromising the effect of NIRS monitoring.

Extremely preterm infants are expected to be included at about 50 NICUs in about 20 countries. The 93 units that took part in a previous funding application for the SafeBoosC III trial had rates of admission of between 15 and 90 extremely preterm infants per year. The total admissions were estimated to be 3000 infants per year. We should, therefore, have a good chance of recruiting 1600 participants within 2 years. Sites that expect to enrol at least 15 participants per year within the 2-year recruitment period will take part. Inclusion of new NICUs after the common start date will be done ad hoc, considering expected contributions and time remaining.

### Randomisation

Infants will be centrally randomised to either the experimental or control group with a 1:1 allocation ratio at the Copenhagen Trial Unit using a web-based randomisation application. The allocation sequence will be computer-generated with varying block sizes concealed for all investigators, as the web-based program will not release the randomisation until the patient has been included in the trial and stratified by NICU and gestational age group (lower gestational age (< 26 weeks) compared to higher gestational age (≥ 26 weeks)). Twin couples will be randomised to the same group, either intervention or control. In centres where only one or two NIRS devices are available, it may not be possible to include all infants from twin births. Thus, only one of a pair of twins may be included. The sibling enrolled will be the one born last.

### Blinding

Due to the nature of the experimental intervention, it is not possible to blind the clinical staff, the infant, or the parents to study group allocation. Outcome assessment of mortality will not be blinded but the mortality data will be checked by Good Clinical Practice via source data verification in all patients. The diagnosis and classification of brain injury along with the entry of these data into the patient report form will be conducted by an assessor blinded to study group allocation. Data entry procedures will depend on local factors and will be agreed on between the principal investigator at each NICU and the coordinating investigator. The data managers, statisticians, and those drawing conclusions will be blinded to study group allocation. Details on this is described in a report on the statistical analysis plan [23].

### Intervention

Experimental group participants will undergo cerebral NIRS monitoring applied as soon as possible after arrival in the NICU and always within 6 hours after delivery and receive treatment based on NIRS monitoring during the first 72 h of life. Treatment will be based on the same evidence-based guideline as used in the SafeBoosC II trial (see below) [24].

The control group participants will not receive any cerebral NIRS monitoring and will be monitored and treated according to local guidelines and clinical practices.

### Treatment guideline based on NIRS monitoring

An evidence-based treatment guideline recommending modification of cardio-respiratory support or interventions aiming at increasing blood oxygen transport capacity will be followed in order to maintain cerebral oxygenation above 55% (Additional file 2) [24]. As the SafeBoosC II trial showed a low burden of hyperoxia unaffected by monitoring-based interventions, the SafeBoosC III trial will not target cerebral hyperoxia and therefore the interventions for hyperoxia have been removed from this trial’s treatment guidelines. The same SafeBoosC III treatment guideline will be used in all participating centres.

### Devices

All commercially available cerebral oximeters that are approved for clinical use in newborns may be used. The aim is to use several different devices to generate results of generic value. There are now seven commercially available devices that are approved for clinical use in different countries: INVOS (Medtronics, Minneapolis, MN, USA); NIRO (Hamamatsu, Hamamatsu City, Japan); Fore-Sight (CAS Medical, Branford, CT, USA); Sensmart (Nonin Medical, Plymouth, MN, USA); O3 (Masimo, Irvine, CA, USA); Egos (Enginmed, Suzhou, China); and Oxyprem 1.4 (Oxyprem, Zürich, Switzerland). The normal range of rStO_2_ was determined with the INVOS adult sensor [17] and defined the rStO_2_ thresholds for intervention used in the SafeBoosC II trial. Each eligible device in SafeBoosC III will be compared with the INVOS adult sensor using a blood lipid phantom and device-specific thresholds will be determined [25] before being used in the SafeBoosC III trial.

### Training and certification

Clinical staff will be offered a web-based training and certification program consisting of short modules covering the trial rationale, NIRS and monitoring of cerebral oxygenation, the treatment guideline, cerebral ultrasound and classification of brain injury, and Good Clinical Practice (www.safeboosc.eu). The use of these modules and the completion rate will be monitored and reported with the results of the trial. Sites with low compliance may be selected for subgroup analyses.

### Trial duration

NIRS monitoring will start within 6 postnatal hours and the intervention will last until 72 h of life. Each participant will be followed up at 36 weeks postmenstrual age.

### Explanatory variables

To allow comparisons between intervention groups, additional baseline clinical data will be obtained, including birth weight, gestational age, mechanical ventilation, and use of cardiovascular support. Data will be drawn from clinical records at 72 h of age and 36 weeks postmenstrual age, the same time as the primary and exploratory outcomes are assessed and documented. The majority of these selected variables are usually reported to neonatal network databases such as the Vermont Oxford Network [26].

### Outcomes

Primary and exploratory outcomes will be assessed at 36 weeks postmenstrual age as documented in the infants’ clinical files. If an infant has been discharged to a step-down unit, data will be sought from that unit, and if this is not possible, data will be used until the date of discharge to the step-down unit. In case the last entry in an infant’s clinical file is prior to 36 + 0 weeks postmenstrual age, for example due to discharge home, the date of discharge will be reported in the online patient report form.

The primary outcome is a composite of either death or severe brain injury detected on any one of a series of cranial ultrasound scans that are routinely performed in extremely premature infants. Severe brain injury is defined as grade III or IV intraventricular haemorrhage (IVH), cystic periventricular leukomalacia (cPVL), cerebellar haemorrhage, post-haemorrhagic ventricular dilatation, or cerebral atrophy. The exploratory outcomes will be bronchopulmonary dysplasia (BPD), retinopathy of prematurity (ROP) stage 3+, necrotising enterocolitis (NEC) stage 2 or higher using the modified Bell’s staging system and/or focal intestinal perforation, late-onset sepsis (> 72 h after birth) defined as being treated with antibiotics for a minimum of 5 days, and a count of the presence of three major neonatal morbidities (BPD, ROP, and severe brain injury). All diagnoses, except severe brain injury, are made as per routine in each NICU.

### Statistical plan and data analysis

Full details regarding statistical considerations and data analysis are outlined in a separate report [23], which will be published before the analysis phase begins, without knowledge of any data collected.

#### Sample size

We have calculated our sample size based on the composite primary outcome, with an alpha of 5%, a power of 90%, and a ratio of experimental trial participants to control trial participants of 1:1.

In the 2009 EuroNeoNet report, the mortality among extremely preterm infants was 33% and severe intracranial haemorrhage was observed in 15%. In the SafeBoosC II trial, the proportion of participants with the composite primary outcome was approximately 34% in the control group and 26% in the experimental group [27].

Based on the above, a total of 1600 infants would be required to demonstrate a similar relative risk reduction of 22%, with an alpha of 5%, and a power of 90%.

In SafeBoosC II, the intra-class correlation coefficient (ICC) of the burden of hypoxia within pairs of twins was negligible. The ICC for death before discharge and for intraventricular haemorrhage grade 3 or 4 have previously been estimated to 0.00 (95% confidence interval (CI) − 0.04 to 0.02) and − 0.01 (95% CI − 0.05 to 0.01) [28]. These values correlate to a design effect very close to 1 [28]. Based on this, we have not included twin ICC in the sample size estimation.

#### Analysis of the primary outcome

The primary outcome analysis will be made on the intention-to-treat population, and we will use mixed-effect logistic regression. ‘Site’ will be included as a random effect (intercept) and the remaining stratification variables, age and intervention groups, will be included as fixed effects. In addition, we will perform a range of pre-defined sensitivity analyses to inform the interpretation of the results of the primary analysis [23].

#### Safety

Predefined serious adverse reactions (SAR) will be reported at 72 h after birth and serious adverse events (SAE) will be reported at 36 weeks postmenstrual age. Expedited reporting will not be used. An independent data monitoring and safety committee is established to monitor mortality, neonatal morbidity, and SARs with ‘certain’ or ‘probably/likely’ relationships with the cerebral NIRS oximeter and/or the application of the evidence-based treatment guideline or any of its interventions. They include two neonatologists and a biostatistician. The charter for the data monitoring and safety committee has been written prior to the enrolment of trial participants. The trial will not be stopped early because of futility, and Lan-DeMets sequential monitoring boundaries will be used at each interim analysis to assess if thresholds for statistical significance of benefits or harms have been crossed [29]. Only one interim analysis is planned, after one-third of trial participants have been randomised. Additional analyses will be decided by the data monitoring and safety committee members [23]. Based on primarily safety considerations, the data monitoring and safety committee will make recommendations to the steering group to continue, change, hold, or terminate the trial. The recommendations will be guided by the statistical monitoring guidelines, which is defined in the data monitoring and safety committee charter (available from www.safeboosc.eu).

The preterm population is at high risk for SAEs and most adverse events may be of a serious nature with or without relevance to the SafeBoosC III trial intervention. Both groups of the trial are expected to have a high proportion of SAEs. It is therefore neither feasible nor meaningful to record and report all adverse events. Therefore, we have decided only to record and report predefined SAEs and SARs. The SAEs include any event of death, severe brain injury, necrotising enterocolitis, bronchopulmonary dysplasia, retinopathy of prematurity, or sepsis as defined under primary and exploratory outcomes. These predefined SAEs have been chosen since they cover the major neonatal morbidities seen in this study population. The SARs are defined as any adverse reaction related to the trial intervention that results in death, is life-threatening, requires prolongation of existing hospitalisation, results in persistent or significant disability or incapacity, or requires intervention to prevent permanent impairment or damage. This includes physical mishaps associated with managing the oximeter and sensors, such as severe skin damage, critical displacement of endotracheal tubes or endovascular lines, and clinical mismanagement based on cerebral oximetry monitoring data, such as interventions aiming at improving cardiovascular status, respiratory status, and/or oxygen transport.

### Data management

All participants’ data are protected in accordance with the Danish Act on the processing of personal data and the Danish Health Act. The Copenhagen Trial Unit will provide central, web-based data entry through an online patient report form, in the open-source clinical trial software OpenClinica®. This will handle the inclusion procedure, the documentation of the stratification and randomisation process, the SARs, and the relevant clinical data from enrolled subjects, including primary and exploratory outcomes and explanatory variables. The data will be entered into the online patient report form directly by the medical staff. Forms for randomisation/inclusion, end-of-monitoring at 72 h of age, and the 36-week follow-up will be created. Data will be stored in accordance with guidelines issued by the Danish Data Protection Agency, from whom approval of the trial will be sought. Only NICU numbers and study numbers will be used to identify participants (i.e. the data kept at Copenhagen Trial Unit is pseudo-anonymised), while lists of study numbers and personal identifying information (e.g. to allow Good Clinical Practice, data cleansing, and later follow-up) will be kept at the NICUs. Six months after the acceptance of the publication that presents the primary outcome, the dataset will be transferred to the Danish data archive. Before transfer, subject study numbers will be removed, NICU numbers will be replaced, sex documentation removed, and birth weight and gestational age recoded into binary variables to minimise the risk of re-identification. Use by other researchers will depend on the permission of the steering group.

The investigators permit trial-related monitoring, audits, and regulatory inspections by providing direct access to the source data and other relevant documents. Trial data will be handled according to regulations of data protection agencies in the respective countries.

### Monitoring

Internal monitoring will be conducted by the Copenhagen Trial Unit, who will monitor patient recruitment and quality, completeness, and timeliness of data entry. In case of problems, the principal investigator will be contacted.

External monitoring will be conducted by a Good Clinical Practice person assigned by the principal investigator at each site. The Good Clinical Practice person will perform monitoring according to the monitoring plan, which will is available at www.safeboosc.eu.

### Ethical considerations

To obtain evidence-based knowledge on the potential benefit and harms of NIRS-based cerebral monitoring in the clinical management of premature infants, large-scale randomised clinical trials are required. The SafeBoosC II trial served as a feasibility trial for the present large-scale SafeBoosC III trial.

In most NICUs, there is still clinical equipoise regarding the use of NIRS monitoring, meaning there is genuine uncertainty over whether cerebral oximetry monitoring and subsequent monitoring-based treatments are clinically beneficial or harmful. Nevertheless, some NICUs have started to use cerebral oxygenation monitoring as part of routine clinical management. Thus, there might be a limited time-window for this trial, since it may be more difficult to test an intervention that is already in clinical use [30]. Therefore, we aim at a pragmatic trial, rather than doing a proof-of-concept trial first.

Extremely preterm infants demonstrate stress reactions during routine manipulation. Positioning and re-positioning of cerebral NIRS sensors can result in such reactions. There are, however, no data to support substantially more risk or discomfort compared with no intervention or compared with current routine care. All interventions proposed in the evidence-based treatment guideline are commonly used in this patient group [21].

‘Treatment as usual’, defined as treatment according to participating hospital’s standard procedures, will be provided to the control group. Also, this will be the care provided to any participant that withdraws consent, in addition to infants who are not included in the trial. Multiple births will be randomised together and undergo allocation to the same study group. This is to avoid parents ascribing differences in their infants’ clinical courses and outcomes based on group allocation resulting from participation in this trial.

### Publication plan

The trial protocol is registered at ClinicalTrials.gov (NCT03770741) and all versions are available at www.safeboosc.eu. Following trial completion, summary trial data will additionally be entered at www.clinicaltrials.gov. Further summary data of main outcomes will be entered after statistical analyses are conducted. Attempts will be made to publish all results, positive, neutral, as well as negative, in a peer-reviewed international journal. Authorship will be determined according to the International Committee of Medical Journal Editors. An additional requirement is one author per NICU completing at least 30 participants. Ancillary studies with results potentially affecting equipoise with regard to the value of NIRS shall not be published before the main publication of the SafeBoosC III trial. After the publication of trial results, depersonalised individual patient data will be uploaded at Zenodo.

## Discussion

In this pragmatic trial, we plan to test the hypothesis that the application of treatment based on cerebral NIRS monitoring in extremely preterm infants will decrease a composite outcome of either death or survival with severe brain injury at 36 weeks postmenstrual age.

A Cochrane systematic review concluded that it is not possible, based on the currently available literature, to determine the specific benefits or harms of NIRS monitoring in extremely preterm infants [27]. The conclusion of this review was that NIRS monitoring should only be used in randomised clinical trials [31]. Despite this, NIRS is routinely used in extremely preterm infants during the first days of life in numerous NICUs in multiple countries [32]. It is likely that this monitoring approach will become more common as evidence in other patient groups becomes more convincing [33]. Therefore, to prevent a non-evidence-based, large-scale clinical uptake of NIRS monitoring, a robust randomised clinical trial, such as the SafeBoosC III trial, is urgently required.

As described in the ‘Blinding’ section, it is not possible to blind the clinical staff, the infants, and the parents of infants participating in this trial. This circumstance introduces risks of bias. Several previous studies have shown that inadequate blinding of participants, personnel, and outcome assessors in randomised trials often results in overestimation of treatment effects for a given intervention for all outcome types, including mortality and subjective outcomes such as radiologic image interpretation [34–37]. A meta-epidemiologic study showed a high variability of treatment effect measured on unblinded subjective outcomes, indicating that for trials including subjective outcomes, the magnitude of bias due to lack of blinding is unpredictable [34]. But again, non-blinded trials compared to similar blinded trials showed overestimation of intervention effects [30]. This meta-epidemiologic study included randomised trials across all clinical fields. A meta-analysis, including 361 intensive-care randomised trials, evaluated the effect of adequate blinding on effect estimates of mortality and found no statistical significant difference between blinded and unblinded trials, suggesting that there may be little, if any, effect of adequate blinding on mortality effect estimates in intensive care trials [38]. No meta-epidemiologic studies, meta-analyses, or systematic reviews have evaluated the effect of adequate/inadequate blinding on intervention effects in neonatal randomised trials. In conclusion, previous results suggest there is a risk of biased results due to lack of blinding even on mortality results. The design of the SafeBoosC III trial strives to minimise the risks regarding the primary outcome.

The pragmatic methodology of this trial also has some limitations. Cranial ultrasound-based diagnoses will be performed locally rather than centrally as was done in SafeBoosC II [18]. This may potentially raise concerns in SafeBoosC III since discrepancies between local readers in different centres could be expected. However, when comparing local and central interpretations of cranial ultrasound images in preterm infants in previous clinical trials, the sensitivity and specificity for local interpretations of severe brain injury were quite robust [39]. Furthermore, we have developed a web-based training program for staff members caring for trial participants. Among other topics, this web program includes a cranial ultrasound module for the purpose of decreasing interobserver variability and heightening data quality.

As in all trial populations of extremely preterm infants, a large number of participants will be twins, which can cause statistical concerns arising from intra-class correlation coefficients (ICC) [28]. We cannot with certainty estimate the ICC for the composite outcome of death or severe brain injury for the present trial. However, the ICC of the burden of hypoxia within pairs of twins in SafeBoosC II was negligible (ICC = 0.027) [27]. Additionally, the twin ICC for pre-discharge death and grade III or IV intraventricular haemorrhage has been estimated in a previous study to 0.00 and − 0.01, which correlates to a negligible design effect [28]. The details of how the twin issue will be statistically accounted for is outlined in the publication of the SafeBoosC III statistical and data analysis plan [23].

The interventions in this trial are complex and rely on a number of separate but interacting components, all relevant for the potential success of the intervention. When NIRS monitors show hypoxic values, neonatologists must evaluate the participant’s clinical status by taking additional measures into consideration and deciding on a possible modification of cardio-respiratory support and interventions to increase blood oxygen transport capacity, based on the treatment guideline. This complexity will result in difficulty interpreting specific results, as it cannot be ascertained what exactly causes a potential effect at 36 weeks postmenstrual age. Furthermore, reproducing and generalising complex interventions may be difficult for future clinicians assessing the results of this trial [40]. However, since this is a pragmatic effectiveness trial evaluating outcomes related to NIRS-based cerebral oxygenation monitoring in routine practice and not the specific treatment choices per se, this concern will not affect the purpose of the trial. The Medical Research Council Framework has developed CONSORT guidelines in order to help trialists develop clearly defined and reproducible complex interventions [41, 42]. We believe that the methodology in the SafeBoosC III trial is in agreement with these guidelines, which is a major strength of this trial.

Obtaining prior informed consent from parents of critically ill neonates within the first hours following birth is difficult and may challenge important standards of information delivery, comprehension, competence, and voluntariness [43–45], and can also restrict the population studied with the effect of impairing the generalisability of results. Furthermore, since monitoring of cerebral oxygenation has been used clinically for several years in other patient groups, and now has entered neonatology to a significant degree, the SafeBoosC III trial can be considered comparative effectiveness research rather than a test of an experimental intervention [46]. Therefore, the protocol allows and encourages principal investigators at each NICU to consider and potentially to seek approval from research ethics boards for one of two other consent forms, i.e. deferred informed consent [43] and prior informed assent (opt-out with enrolment as default) [47]. We believe this offers appropriate flexibility in an international trial in an area where legitimate ethical considerations are in conflict. For this purpose, we have developed parental information sheets specific for each consent method (Additional file 3).

Though extremely preterm infants constitute only 0.5% of all births [1], they represent an extremely high-risk population, and thus their contribution to infant mortality and to the prevalence of cerebral palsy exceeds 10% [48–50]. Accumulating evidence indicates that cerebral hypoxia is a significant cause of mortality as well as brain injury in this population. Thus, monitoring of cerebral oxygenation levels during the first days after birth has the potential to address a significant health problem. Although the overall risk in this population is high, there are many other relevant contributing factors to mortality and brain injury, and thus only a moderate risk reduction can be expected. Therefore, a trial to address this therapeutic question must be large in scope. If the experimental intervention proves successful, we may save 2000 extremely preterm infants or more every year from death or a life with handicap due to brain injury in high-income countries. The ensuing health economics impact may thus be quite robust.

In conclusion, there is an urgent need for a randomised clinical trial to assess the effects of cerebral NIRS monitoring compared with treatment as usual in extremely preterm infants.

## Trial status

The protocol is registered at www.clinicaltrials.gov (NCT03770741; registered 10 December 2018). The first infant was enrolled in June 2019 and the anticipated date of study completion is October 2021. Recruitment status can be accessed at www.safeboosc.eu.

## Supplementary information

**Additional file 1.** SPIRIT checklist.

**Additional file 2.** Treatment guideline. A description of the treatment guideline that will be used in the SafeBoosC III trial.

**Additional file 3.** Parental information sheets and consent form. Templates for parental information sheets for different consent methods and a general consent form.

**Additional file 4.** WHO trial registration data set.

## Data Availability

Not applicable.

## Abbreviations

BPD: Bronchopulmonary dysplasia
cPVL: Cystic periventricular leukolamacia
ICC: Intra-class correlation coefficients
IVH: Intraventricular haemorrhage
NEC: Necrotizing enterocolitis
NICU: Neonatal intensive care unit
NIRS: Near-infrared spectroscopy
ROP: Retinopathy of prematurity
SAE: Severe adverse events
SAR: Severe adverse reactions
