# Supplementary material for: Cerebral near-infrared spectroscopy monitoring versus treatment as usual for extremely preterm infants: a protocol for the SafeBoosC randomised clinical phase III trial
Source: Trials. 2019 Dec 30;20:811. doi: 10.1186/s13063-019-3955-6 (PMC6937938; doi:10.1186/s13063-019-3955-6)
Supplement: Supplementary file 2 — Additional file 2. Treatment guideline. A description of the treatment guideline that will be used in the SafeBoosC III trial. [file 13063_2019_3955_MOESM2_ESM.docx]

## Additional file 2: Evidence-based treatment guidelines and justifications

**SafeBoosC-III Clinical Guidelines**

**Assessment of cerebral oxygen saturation**

Regional cerebral tissue oxygen saturation (rStO_2_) is a composite measure of tissue oxygen saturation across arterial, capillary and venous beds and reflects a balance between cerebral oxygen delivery (CDO_2_) and cerebral metabolic rate (CMRO_2_). In preterm infants, the CMRO_2_ is unlikely to vary much and a change in rStO_2_ largely reflects changes in CDO_2_. The factors which influence CDO_2_ are arterial oxygen saturation (SaO_2_), haemoglobin concentration and cerebral blood flow (CBF).

**Establishment of monitoring of cerebral oxygenation**

As soon as possible and within 6 hours of age

**Period of monitoring of cerebral oxygenation**

Until 72 hours after birth

**Recommendation for clinical interventions**

The thresholds for intervention depends on the oximeter. If StO2 is predominantly below the hypoxic threshold over a 10-minute period or drops acutely and markedly under the threshold, the sensor should be inspected for any potential displacement, and possibly be repositioned. If this does not solve the problem, a decision regarding intervention (modification of cardio-respiratory support) should be made (identified in ‘•’) as listed below and StO_2_ reassessed 30 to 60 minutes after the intervention. Generally, only one intervention should be chosen at a time. All the interventions proposed here are commonly used in this patient group.

For each intervention, the level of evidence (I-III) and strength of recommendation (A-E) are given (defined in Tables 1 and 2). For further explanation, see below.

Rationale/aim of interventions: A low rStO_2_ reflects a low CDO_2_. The interventions should be directed to increasing CBF, blood haemoglobin concentration, or SaO_2_.

Assess cardiovascular status:

Blood pressure below the normal range or low, even in the normal range, consider:

- Vasopressor-inotropes (I/B) (64,65)
- Fluid bolus (normal saline) (I/C) (66,67)
- Decrease mean airway pressure on ventilator or CPAP (III/B) (68–71)

Poor systemic circulation, consider if:

Echocardiography shows low cardiac output and/or low SVC flow, consider:

- Inotropes (I/B) (31,67,72–75)
- Fluid bolus (normal saline) (I/C) (66,67)
- Decrease mean airway pressure (III/B) (68–71)
- Reduce vasopressor (III/ B) (76)

**Echocardiography not available but has at least 2 of the following signs:**

Lactate > 3.5 mmol/l

Capillary Refill Time > 3 seconds

Urine output < 1 ml/kg/hour

consider:

- Inotropes (I/B) (31,67,72–75)
- Fluid bolus (normal saline) (I/C) (66,67)
- Decrease mean airway pressure (III/B) (68–71)
- Reduce vasopressor (III/ B) (76)

**Patent ductus arteriosus, consider:**

- Medical treatment (II-2/B) (69,70,77,78)

Assess oxygen transport:

Blood haemoglobin concentration below the normal range or low, even in the normal range, consider:

Red blood cell transfusion (I/B) (79–82)

Assess respiratory status:

SaO2 below the normal range or low, even in normal range, consider:

- Increase FiO_2_ (II-1/A) (83) (ATTENTION: be careful not to exceed the upper target threshold of SpO_2_)
- Increase mean airway pressure (III/B) (68,84,85)

PCO_2_ below the normal range or low, even in normal range, consider:

- Decrease minute ventilation - (II/A) (79,86–88)

Level of evidence and recommendation of intervention

The level of evidence (Table 1) and recommendation for a given intervention (in brackets and Table 2) were graded according to the U.S. Preventive Services Task Force system (89)

**Table 1:** Hierarchy of research design and level of evidence

| **Level of evidence** | **Type of study** |
| --- | --- |
| I | Evidence obtained from at least one properly randomized controlled trial |
| II-1 | Evidence obtained from well-designed controlled trials without randomization |
| II-2 | Evidence obtained from well-designed cohort or case-control analytic studies, preferably from more than one centre or research group |
| II-3 | Evidence obtained from multiple time series with or without intervention. Dramatic results in uncontrolled experiments (such as the results of the introduction of penicillin treatment in the 1940s) could also be regarded as this type of evidence |
| III | Opinions of respected authorities, based on clinical experience, descriptive studies and case reports, or reports of expert committees |

**Table 2:** Recommendation grid

| **Net benefit** | | | | |
| --- | --- | --- | --- | --- |
| **Quality of evidence** | substantial | moderate | small | zero/negative |
| Good | A | B | C | D |
| Fair | B | B | C | D |
| Poor | E | E | E | E |
| Standard recommendation language | A= Strongly recommended (good evidence that the intervention improves important health outcomes and benefits substantially outweigh harms).  B= Recommended (at least fair evidence that the intervention improves important health outcomes and benefits substantially outweigh harms).  C= No recommendation for or against routine provision of the intervention (fair evidence that the service can improve health outcomes but the balance of the benefits and harms is too close to justify a general recommendation).  D= Recommends against routinely providing the intervention (at least fair evidence that the service is ineffective or that harms outweigh benefits).  E= Insufficient to recommend for or against routinely providing the intervention (evidence that the intervention is effective is lacking, of poor quality, or conflicting and the balance of benefits and harms cannot be determined). | | | |

**Abbreviations**

| SVC | Superior vena cava |
| --- | --- |
| CRT | Capillary refill time |
| FiO_2_ | Fraction of inspired oxygen |
| rStO_2_ | Regional cerebral tissue oxygen saturation |
| SaO_2_ | Saturation of oxygen (arterial blood) |
| PCO_2_ | Partial pressure of carbon dioxide |
| CDO_2_ | Cerebral oxygen delivery |
| CMRO_2_ | Cerebral metabolic rate |
| CBF | Cerebral blood flow |
|  |  |
