# Supplementary material for: Cerebral near-infrared spectroscopy monitoring versus treatment as usual for extremely preterm infants: a protocol for the SafeBoosC randomised clinical phase III trial
Source: Trials. 2019 Dec 30;20:811. doi: 10.1186/s13063-019-3955-6 (PMC6937938; doi:10.1186/s13063-019-3955-6)
Supplement: Supplementary file 3 — Additional file 3. Parental information sheets and consent form. Templates for parental information sheets for different consent methods and a general consent form. [file 13063_2019_3955_MOESM3_ESM.docx]

**Additional file 3: parental information sheets and consent form**

Deferred informed consent – parental information for control group participants

*Information to parents who have given birth to a baby born very preterm and has been included in the SafeBoosC trial.*

*Regarding the trial****:*** **SafeBoosC-III: Safeguarding the brain of our smallest children**

*An investigator-initiated, pragmatic, open label, multinational randomized phase III clinical trial evaluating treatment based on near-infrared spectroscopy monitoring versus treatment as usual in premature infants.*

***Dear Parent***

You are receiving this leaflet because your baby has been born very early and needs intensive care. We understand that this may be a stressful time for you and that it can be difficult to take in information at this time. This leaflet gives you information about a clinical trial called SafeBoosC-III. We are conducting this trial to investigate if monitoring of the brain’s oxygen levels can optimize intensive care treatment and thereby, improve the care of babies in the same situation as yours.

Because the trial must begin within the first six hours of life, we have already randomised your baby. Your baby was randomised to the control group (see below in ‘What does trial participation involve”). We now ask for your permission to continue having your baby in the trial. You are free to decide.

***What does trial participation involve?***

If a baby gets randomised, i.e. is randomly selected, to the control group, as is the case for your baby, he or she will receive the usual treatment and support given in the neonatal intensive care unit. This means he/she will still be given the highest level of care and attention by staff.

If, on the other hand, a baby gets randomised to the experimental group, he or she will also have a small sensor put on the head. As mentioned earlier, this has to be done within six hours after birth. The sensor will be in place until 72 hours after birth. If the sensor shows that there may be too little oxygen in the brain, the doctor may adjust the support of the baby’s breathing and circulation to try to improve it.

There are no extra examinations done, but data from your baby’s hospital records will be used to calculate the result of the trial. The trial is multinational and has also been approved by an ethics review board in your country.

***What are we trying to find out?***

We want to know if monitoring of the brain’s oxygenation (keeping an eye on the amount of oxygen in the brain) can increase the chance of survival and decrease the risk of brain injury. The trial is based on results from a previous trial, SafeBoosC-II, where monitoring of brain oxygenation appeared to be beneficial. However, we need to test this on a larger scale. If it is successful, it may help prevent death or a life with handicap, for at least 2,000 babies born much too early every year world-wide.

***This is the essential information. On the next pages you can read more details and read the standard conditions for participation in research projects. If you want to know more you can find the full protocol and other information about the trial on*** [***www.safeboosc.eu***](http://www.safeboosc.eu)***. Do not hesitate to ask questions.***

***In the following pages you can read more details about the SafeBoosC-III trial.***

***How can we know the oxygenation of the brain?***

We use an instrument called a near-infrared oximeter. It has a thin cable attached to a sensor (a small, soft patch). The sensor is put on the head of the baby and held in place by a bandage. The sensor uses near-infrared light. The light goes a few centimetres into the brain and measures the colour of the red blood cells as it changes with oxygenation. The oximeter does that every 5 seconds and the value of oxygenation is shown on the oximeter at all times.

***How will this change the treatment of my baby?***

If the oximeter shows a low value, the doctor will adjust the treatment and support that your baby receives. The adjustments follow a guideline that lists the ways treatment may be used to respond to a low oxygen in the brain. These treatments are all used in routine clinical practice in order to support respiration, blood circulation, and blood transport of gasses (oxygen, carbondioxide). The only addition now is that the use of treatments is guided also by the oxygen levels in the brain.

***How will the research be done?***

SafeBoosC-III is a randomised clinical trial. Many treatments are tested by randomised clinical trials, as it is the most reliable way to find out if they are effective.

A randomised clinical trial means that the decision about whether the baby will receive monitoring of brain oxygenation will be determined by chance, like the toss of a coin. This means that half of the babies participating in the SafeBoosC-III trial will receive treatment guided by monitoring of the brain oxygenation (experimental group) while the other half will receive treatment as usual, i.e. no monitoring of the brain (control group). We need 1600 babies from approximately 50 hospitals in Europe, USA and China to evaluate the intervention effect.

***Are there risks?***

Near-infrared light is difficult to see but can go some centimetres into the body. It is more like heat than light. The heat is less than that from the sun on a normal summer day and thus, it has no risks to the brain. There is, however, a small risk of skin burns, like on a sunny day. In the previous trial, SafeBoosC II, skin marks were seen in 10% of babies. In very rare cases this may lead to a scar. This risk can be minimised by moving the sensor at intervals. The nurse will do this very carefully in order not to disturb your baby.

We do the trial because with think that the specific guidelines (the adjustments to treatment) will prove beneficial to the baby, i.e. increase the chance of survival and reduce the risk of brain injury. It is already in routine use in neonatal departments, for instance in Holland, the US and Finland. When a baby is born too early, however, there is a risk that he/she may die or suffer damage to the brain, lungs, intestines or eyes. General experience shows that new interventions may be better in some ways and yet can cause an increased risk in other ways. For the SafeBoosC-III trial it could reduce the risk of death and be better for the brain but worse for the lungs or the eyes. We do not know at present. That is why it is necessary to test it properly in a randomised trial to clarify risks and benefits before taking it into routine use. So, as in all research, there may be unforeseen risks. All severe adverse reactions will be reported as a part of the research to make sure that we learn as much as possible from it.

All observations, treatments and additional monitoring will be as usual in the neonatal department.

The investigators will access your baby’s hospital records for trial purposes, i.e. trial completion, surveillance and control of trial.

Any information that we collect from the hospital records will be kept confidential and in a secure place. A pseudoanonymised copy (identified only by a study number, so your child cannot be identified) will be sent to the trial centre in Copenhagen for statistics. Only authorised people involved in the trial will have access to data from your baby.

***Follow-up studies***

We are planning to roll out an additional study to follow up on your baby’s progress. Therefore, you may be contacted within the first two years of your baby’s life. Follow up may include a doctor’s examination, a questionnaire or a developmental test. You will be asked for separate consent for this or any other study that may be relevant for babies that take part in the SafeBoosC trial.

***Does my baby have to continue in SafeBoosC-III?***

No. It is your decision whether or not your baby should continuet in the SafeBoosC-III trial. If you decide to let your baby continue in the trial, you may change your mind later without having to give a reason and without it having any consequences to your baby's future care in any way.

If you decide that you do not want your baby to continue in the SafeBoosC-III trial, he or she will still be given the highest level of care and attention by staff.

***Exclusion from or termination of the trial***

If the cerebral oximetry monitoring causes unexpected problems for your baby, it will be stopped. With your permission, we will still want to include the data of your baby in the evaluation of the trial.

The trial is monitored by an external committee. If it appears that there are important unexpected problems or it appear that the trial will not be able to answer the questions that it was designed to answer, the trial may be stopped.

***Will I be told the results of SafeBoosC if we join?***

Yes. If you decide that you would like your baby to join SafeBoosC-III we will keep in touch with you to tell you the results of the trial when they become available (in year 2021-22), if you wish so.

The results from the SafeBoosC trial will, regardless of positive, negative, or inconclusive, be published in a public accessible journal.

***What will happen to the data you have collected regarding my baby?***

The persons involved in this trial have no economic interest in any aspects of the trial. This trial is solely made to improve the care of babies born too early. Therefore, in order to optimize the value of your baby’s participation, we will store your baby’s anonymized data for at least 10 years so that other researchers may use them to examine other research questions in the same field.

***Funding for the trial***

The trial was planned by our group of European, academic neonatologists and funded by the Elsass Foundation of Denmark with a total amount of 0.4 mio euro to cover the central trial costs. We are applying for further funding at the Medtronics Foundation and the Novo Nordisk Foundation.

The trial sponsor Prof. Gorm Greisen or any other investigator in the trial, has no personal attachment to Elsass Foundation, Medtronics Foundation, Novo Nordisk Foundation or any other organisation/company with interest in near-infrared spectroscopy monitoring.

Families will not be payed a fee for participation.

***The SafeBoosC-III trial has been authorised by (insert name of research ethics committee)***

***Thank you***

Thank you for taking the time to read this leaflet. If you would like more information about SafeBoosC-III, please ask your doctor or nurse.

**Contact Information the SafeBoosC-III trial**

**Sponsor:** Prof. Gorm Greisen

Address: Neonatalklinikken GN, Blegdamsvej 9, 2100 København Ø, Denmark

Telephone: +45 35 45 43 20

**Local contact person**

Address:

Telephone:

Deferred informed consent – parental information for experimental group participants

*Information to parents who have given birth to a baby born very preterm and has been included in the SafeBoosC trial.*

*Regarding the trial****:*** **SafeBoosC-III: Safeguarding the brain of our smallest children**

*An investigator-initiated, pragmatic, open label, multinational randomized phase III clinical trial evaluating treatment based on near-infrared spectroscopy monitoring versus treatment as usual in premature infants.*

***Dear Parent***

You are receiving this leaflet because your baby has been born very early and needs intensive care. We understand that this may be a stressful time for you and that it can be diificult to take in information at this time. This leaflet gives you information about a clinical trial called SafeBoosC-III. We are conducting this trial to investigate if monitoring of the brain’s oxygen levels can optimize intensive care treatment and thereby, improve the care of babies in the same situation as yours.

Because the trial must begin within the first six hours of life, we have already started measuring your baby’s oxygen level in the brain through a small exterior sensor. In clinical words, we randomised your baby to the experimental group. We now ask for your permission to continue having your baby in the trial. You are free to decide.

***What does trial participation involve?***

If a baby gets randomised, i.e. is randomly selected, to the control group, he or she will receive the usual treatment and support given in the neonatal intensive care unit. This means he/she will still be given the highest level of care and attention by staff.

If, on the other hand, a baby gets randomised to the experimental group, as is the case for your baby, he or she will also have a small sensor put on the head. As mentioned earlier, this has to be done within six hours after birth. The sensor will be in place until 72 hours after birth. If the sensor shows that there may be too little oxygen in the brain, the doctor may adjust the support of the baby’s breathing and circulation to try to improve it.

There are no extra examinations done, but data from your baby’s hospital records will be used to calculate the result of the trial. The trial is multinational and has also been approved by an ethics review board in your country.

***What are we trying to find out?***

We want to know if monitoring of the brain’s oxygenation (keeping an eye on the amount of oxygen in the brain) can increase the chance of survival and decrease the risk of brain injury. The trial is based on results from a previous trial, SafeBoosC-II, where monitoring of brain oxygenation appeared to be beneficial. However, we need to test this on a larger scale. If it is successful, it may help prevent death or a life with handicap for at least 2,000 babies born much too early every year world-wide.

***This is the essential information. On the next pages you can read more details and read the standard conditions for participation in research projects. If you want to know more you can find the full protocol and other information about the trial on*** [***www.safeboosc.eu***](http://www.safeboosc.eu)***. Do not hesitate to ask questions.***

***In the following pages you can read more details about the SafeBoosC-III trial.***

***How can we know the oxygenation of the brain?***

We use an instrument called a near-infrared oximeter. It has a thin cable attached to a sensor (a small, soft patch). The sensor is put on the head of the baby and held in place by a bandage. The sensor uses near-infrared light. The light goes a few centimetres into the brain and measures the colour of the red blood cells as it changes with oxygenation. The oximeter does that every 5 seconds and the value of oxygenation is shown on the oximeter at all times.

***How will this change the treatment of my baby?***

If the oximeter shows a low value, the doctor will adjust the treatment and support that your baby receives. The adjustments follow a guideline that lists the ways treatment may be used to respond to a low oxygen in the brain. These treatments are all used in routine clinical practice in order to support respiration, blood circulation, and blood transport of gasses (oxygen, carbondioxide). The only addition now is that the use of treatments is guided also by the oxygen levels in the brain.

***How will the research be done?***

SafeBoosC-III is a randomised clinical trial. Many treatments are tested by randomised clinical trials, as it is the most reliable way to find out if they are effective.

A randomised clinical trial means, that the decision about whether the baby will receive monitoring of brain oxygenation will be determined by chance, like the toss of a coin. This means that half of the babiesparticipating in the SafeBoosC-III trial will receive treatment guided by monitoring of the brain oxygenation (experimental group) while the other half will receive treatment as usual, i.e. no monitoring of the brain (control group). We need 1600 babies from approximately 50 hospitals in Europe, USA and China to evaluate the intervention effect.

***Are there risks?***

Near-infrared light is difficult to see but can go some centimetres into the body. It is more like heat than light. The heat is less than that from the sun on a normal summer day and thus, it has no risks to the brain. There is, however, a small risk of skin burns, like on a sunny day. In the previous trial, SafeBoosC-II skin marks were seen in 10% of babies. In very rare cases this may lead to a scar. This risk can be minimised by moving the sensor at intervals. The nurse will do this very carefully in order not to disturb your baby.

We do the trial because with think that the specific guidelines (the adjustments to treatment) will prove beneficial to the baby, i.e. increase the chance of survival and reduce the risk of brain injury. It is already in routine use in neonatal departments, for instance in Holland, the US and Finland. When a baby is born too early, however, there is a risk that he/she may die or suffer damage to the brain, lungs, intestines or eyes. General experience shows that new interventions may be better in some ways and yet can cause an increased risk in other ways. For the SafeBoosC-III trial it could reduce the risk of death and be better for the brain but worse for the lungs or the eyes. We do not know at present. That is why it is necessary to test it properly in a randomised trial to clarify risks and benefits before taking it into routine use. So, as in all research, there may be unforeseen risks. All severe adverse reactions will be reported as a part of the research to make sure that we learn as much as possible from it.

All observations, treatments and additional monitoring will be as usual in the neonatal department. In particular, ultrasound scanning of the brain will be conducted several times as part of normal practice.

The investigators will access your baby’s hospital records for trial purposes, i.e. trial completion, surveillance and control of trial.

Any information that we collect from the hospital records will be kept confidential and in a secure place. A pseudoanonymised copy (identified only by a study number, so your child cannot be identified) will be sent to the trial centre in Copenhagen for statistics. Only authorised people involved in the trial will have access to data from your baby.

***Follow-up studies***

We are planning to roll out an additional study to follow up on your baby’s progress. Therefore, you may be contacted within the first two years of your baby’s life. Follow up may include a doctor’s examination, a questionnaire or a developmental test. You will be asked for separate consent for this or any other study that may be relevant for babies that take part in the SafeBoosC trial.

***Does my baby have to continue in SafeBoosC-III?***

No. It is your decision whether or not your baby should continue in the SafeBoosC-III trial. If you decide to let your baby continue in the trial, you may change your mind later without having to give a reason and without it having any consequences to your baby's future care in any way.

If you decide that you do not want your baby to continue in the SafeBoosC-III trial, he or she will still be given the highest level of care and attention by staff.

***Exclusion from or termination of the trial***

If the cerebral oximetry monitoring causes unexpected problems for your baby, it will be stopped. With your permission, we will still want to include the data of your baby in the evaluation of the trial.

The trial is monitored by an external committee. If it appears that there are important unexpected problems, or it appears that the trial will not be able to answer the questions that it was designed to answer, the trial may be stopped.

***Will I be told the results of SafeBoosC if we join?***

Yes. If you decide that you would like your baby to join SafeBoosC-III we will keep in touch with you to tell you the results of the trial when they become available (in year 2021-22),if you wish so.

The results from the SafeBoosC trial will, regardless of positive, negative, or inconclusive, be published in a public accessible journal.

***What will happen to the data you have collected regarding my baby?***

The persons involved in this trial have no economic interest in any aspects of the trial,. This trial is solely made to improve the care of babies born too early. Therefore, in order to optimize the value of your baby’s participation, we will store your baby’s anonymized data fro at least 10 years so that other researchers may use them to examine other research questions in the same field.

***Funding for the trial***

The trial was planned by our group of European, academic neonatologists and funded by the Elsass Foundation of Denmark with a total amount of 0.4 mio euro to cover the central trial costs.

We are applying for further funding at the Medtronics Foundation and the Novo Nordisk Foundation.

The trial sponsor Prof. Gorm Greisen or any other investigator in the trial, has no personal attachment to Elsass Foundation, Medtronics Foundation, Novo Nordisk Foundation or any other organisation/company with interest in near-infrared spectroscopy monitoring.

Families will not be payed a fee for participation.

***The SafeBoosC-III trial has been authorised by (insert name of research ethics committee)***

***Thank you***

Thank you for taking the time to read this leaflet. If you would like more information about SafeBoosC-III, please ask your doctor or nurse.

**Contact Information the SafeBoosC-III trial**

**Sponsor:** Prof. Gorm Greisen

Address: Neonatalklinikken GN, Blegdamsvej 9, 2100 København Ø, Denmark

Telephone: +45 35 45 43 20

**Local contact person**

Address:

Telephone:

Prior assent – parental information for postnatal recruitment

*Information to parents who have given birth to a baby extremely preterm*

*Regarding the trial****:*** **SafeBoosC-III: Safeguarding the brain of our smallest children**

*An investigator-initiated, pragmatic, open label, multinational randomized phase III clinical trial evaluating treatment based on near-infrared spectroscopy monitoring versus treatment as usual in premature infants.*

***Dear Parent***

You are receiving this leaflet because your baby has been born very early and needs intensive care. We understand that this may be a stressful time for you and that it can be hard to take in information at this time. This leaflet gives you information about a randomised clinical trial called SafeBoosC-III. We are conducting this trial to investigate if monitoring of the brain’s oxygen levels can optimize intensive care treatment and thereby, improve the care of babies in the same situation as yours.

The trial tests the value of using an optical sensor to monitor the oxygenation of the brain in the first days of life. This is already routinely used in preterm infants in hospitals, for instance in Holland, USA and Finland, but other hospitals do not use it due to lack of research within the field. It is also commonly used in other patients, such as during heart surgery and in the days after. Trial participation do not involve any trial-related extra procedures. Due to the urgency of this trial, we need to start as soon as possible after birth. Therefore, your baby will be included in the trial and randomised to either the experimental or control group, unless you decide not to let your baby participate. If you do not want your baby to participate, please inform a doctor or a nurse as soon as possible. After having explained and discussed the information in this leaflet with you, the doctor will record in your child’s clinical file that he/she has explained this to you and whether or not you decide to opt-out, i.e. not let your child continue in the trial. Your decision on this will not influence the care of your baby in any other way.

***On the next page there is a short text with more details.***

***What does trial participation involve?***

If your baby gets randomised to the control group he or she will just receive the usual treatment and support given in the neonatal intensive care unit.

If, on the other hand, your baby gets randomised to the experimental group, he or she will also have a small sensor put on the head. This has to be done within three hours after birth. The sensor will be in place until 72 hours after birth. If the sensor shows that there may be too little oxygen in the brain, the doctor may adjust the support of the baby’s breathing and circulation to try to improve it.

There are no extra examinations done, but data from your baby’s hospital records will be used to calculate the result of the trial. The trial is multinational and has also been approved by an ethics review board in your country.

***What are we trying to find out?***

We want to know if monitoring of brain oxygenation (keeping an eye on the amount of oxygen in the brain) can increase the chance of survival and decrease the risk of brain injury. The trial is based on results from a previous trial, SafeBoosC-II, where monitoring of brain oxygenation appeared to be beneficial. However, we need to test this on a larger scale. If it is successful, it may lead to the saving of at least 2,000 babies born much too early every year from death or a life with handicap world-wide.

***This is the essential information. On the next pages you can read more details and read the standard conditions for participation in research projects. If you want to know more you can find the full protocol and other information about the trial on*** [***www.safeboosc.eu***](http://www.safeboosc.eu)***. Also, do not hesitate to ask questions.***

***In the following pages you can read more details about the SafeBoosC-III trial.***

***How can we know the oxygenation of the brain?***

We use an instrument called a near-infrared oximeter. It has a thin cable attached to a sensor (a small, soft patch). The sensor is put on the head of the baby and held in place by a bandage. The sensor uses near-infrared light. The light goes a few centimetres into the brain and measures the colour of the red blood cells as it changes with oxygenation. The oximeter does that every 5 seconds and the value of oxygenation is shown on the oximeter at all times.

***How will this change the treatment of my baby?***

If the oximeter shows a low value, the doctor will adjust the treatment and support that your baby receives. The adjustments follow a guideline that lists the ways treatment may be used to respond to a low oxygen in the brain. These treatments are all used in routine clinical practice in order to support respiration, blood circulation, and blood transport of gasses (oxygen, carbondioxide). The only addition now is that the use of treatments is guided also by the oxygen levels in the brain.

***How will the research be done?***

SafeBoosC-III is a randomised clinical trial. Many treatments are tested by randomised clinical trials, as it is the most reliable way to find out if they are effective.

A randomised clinical trial means that the decision about whether the baby will receive monitoring of brain oxygenation will be determined by chance, like the toss of a coin. This means that half of the babies that will participate in the SafeBoosC-III trial will receive treatment guided by monitoring of the brain oxygenation (experimental group) while the other half will receive treatment as usual, i.e. no monitoring of the brain (control group). We need 1600 babies from approximately 50 hospitals in Europe, USA and China to evaluate the intervention effect.

***Are there risks?***

Near-infrared light is difficult to see but can go some centimetres into the body. It is more like heat than light. The heat is less than that from the sun on a normal summer day and thus, it has no risks to the brain. There is, however, a small risk of skin burns, like on a sunny day. This risk can be minimised by moving the sensor at intervals. The nurse will do this very carefully in order not to disturb the child.

We hope that the specific guidelines (the adjustments to treatment) will prove beneficial to the baby, i.e. increase the chance of survival and reduce the risk of brain injury. When a baby is born too early, there is a risk that it may die or suffer damage to the brain, lungs, intestines or eyes. What we test in this trial is a new type of monitoring which is increasingly being used in neonatal departments, for instance in Holland, USA and Finland. General experience shows that new interventions may be better in some ways and yet can cause an increased risk in other ways. For the SafeBoosC-III trial it could be better for the brain but worse for the lungs or the eyes. We do not know at present. That is why it is so important to do randomised trials to clarify risks and benefits. So, as in all research, there may be unforeseen risks. All unexpected adverse events will be reported as a part of the research.

All observations, monitoring, and treatments will be as usual in the neonatal department. In particular, ultrasound scanning of the brain will be conducted several times as part of normal practice.

The investigators will access your baby’s hospital records for trial purposes, i.e. trial completion, surveillance and control of trial.

Any information that we collect from the hospital records will be kept confidential and in a secure place. A pseudoanonymised copy (identified only by a study number) will be sent to the trial centre in Copenhagen for statistics. Only authorised people involved in the trial will have access to data from your baby.

***Ancillary studies***

We are planning to roll out an ancillary study to follow up on your baby’s progress. Therefore, you may be contacted within the first two years of your baby’s life. Follow up may include a doctor’s examination, a questionnaire or a developmental test. You will be asked for separate consent for this or any other study that may be relevant for babies that take part in the SafeBoosC trial.

***Does my baby have to take part in SafeBoosC-III?***

No. It is your decision whether or not your baby should take part in the SafeBoosC-III trial. If you decide not to ‘opt-out’, you may change your mind later without having to give a reason and without it having any consequences to your baby's future care in any way.

If you decide that you do not want your baby to join the SafeBoosC-III trial at all, he or she will still be given the highest level of care and attention by staff.

***Exclusion from or termination of the trial***

If the cerebral oximetry monitoring causes unexpected problems for your baby, it will be stopped. With your permission, we will still want to include the data of your baby in the evaluation of the trial.

The trial is monitored by an external committee. If it appears that there are important unexpected problems or it appear that the trial will not be able to answer the questions that it was designed to answer, the trial may be stopped.

***Will I be told the results of SafeBoosC if we join?***

Yes. If you decide that you would like your baby to join SafeBoosC-III we will keep in touch with you to tell you the results of the trial when they become available (in year 2021-22), if you wish so.

The results from the SafeBoosC trial will, regardless of positive, negative, or inconclusive, be published in a public accessible journal.

***Funding for the trial***

The trial was planned by our group of European, academic neonatologists and funded by the Elsass Foundation of Denmark with a total amount of 0.4 mio euro to cover the central trial costs.

The trial sponsor Prof. Gorm Greisen or any other investigator in the trial, has no personal attachment to Elsass Foundation or any other organisation/company with interest in near-infrared spectroscopy monitoring.

Families will not be payed a fee for participation.

***The SafeBoosC-III trial has been authorised by (insert name of research ethics committee)***

***Thank you***

Thank you for taking the time to read this leaflet. If you would like more information about SafeBoosC-III, please ask your doctor or nurse.

**Contact Information the SafeBoosC-III trial**

**Sponsor:** Prof. Gorm Greisen

Address: Neonatalklinikken GN, Blegdamsvej 9, 2100 København Ø, Denmark

Telephone: +45 35 45 43 20

**Local contact person**

Address:

Telephone:

‘

Prior assent – parental information for prenatal recruitment

*Information to parents who may give birth to a baby extremely preterm*

*Regarding the trial****:*** **SafeBoosC-III: Safeguarding the brain of our smallest children**

*An investigator-initiated, pragmatic, open label, multinational randomized phase III clinical trial evaluating treatment based on near-infrared spectroscopy monitoring versus treatment as usual in premature infants.*

***Dear Parent***

You are receiving this leaflet because your baby may been born very early and may needs intensive care. We understand that this may be a stressful time for you and that it can be hard to take in information at this time. This leaflet gives you information about a randomised clinical trial called SafeBoosC-III. We are conducting this trial to investigate if monitoring of the brain’s oxygen levels can optimize intensive care treatment and thereby, improve the care of babies in the same situation as yours.

The trial tests the value of using an optical sensor to monitor the oxygenation of the brain in the first days of life. This is already routinely used in preterm infants in hospitals, for instance in Holland, USA and Finland, but other hospitals do not use it due to lack of research within the field. It is also commonly used in other patients, such as during heart surgery and in the days after. Trial participation do not involve any trial-related extra procedures. Due to the urgency of this trial, we need to start as soon as possible after birth. Therefore, your baby will be included in the trial and randomised to either the experimental or control group, unless you decide not to let your baby participate. If you do not want your baby to participate, please inform a doctor or a nurse as soon as possible. After having explained and discussed the information in this leaflet with you, the doctor will record in your child’s clinical file that he/she has explained this to you and whether or not you decide to opt-out, i.e. not let your child continue in the trial. Your decision on this will not influence the care of your baby in any other way.

***On the next page there is a short text with more details.***

***What does trial participation involve?***

If your baby gets randomised to the control group he or she will just receive the usual treatment and support given in the neonatal intensive care unit.

If, on the other hand, your baby gets randomised to the experimental group, he or she will also have a small sensor put on the head. This has to be done within three hours after birth. The sensor will be in place until 72 hours after birth. If the sensor shows that there may be too little oxygen in the brain, the doctor may adjust the support of the baby’s breathing and circulation to try to improve it.

There are no extra examinations done, but data from your baby’s hospital records will be used to calculate the result of the trial. The trial is multinational and has also been approved by an ethics review board in your country.

***What are we trying to find out?***

We want to know if monitoring of brain oxygenation (keeping an eye on the amount of oxygen in the brain) can increase the chance of survival and decrease the risk of brain injury. The trial is based on results from a previous trial, SafeBoosC-II, where monitoring of brain oxygenation appeared to be beneficial. However, we need to test this on a larger scale. If it is successful, it may lead to the saving of at least 2,000 babies born much too early every year from death or a life with handicap world-wide.

***This is the essential information. On the next pages you can read more details and read the standard conditions for participation in research projects. If you want to know more you can find the full protocol and other information about the trial on*** [***www.safeboosc.eu***](http://www.safeboosc.eu)***. Also, do not hesitate to ask questions.***

***In the following pages you can read more details about the SafeBoosC-III trial.***

***How can we know the oxygenation of the brain?***

We use an instrument called a near-infrared oximeter. It has a thin cable attached to a sensor (a small, soft patch). The sensor is put on the head of the baby and held in place by a bandage. The sensor uses near-infrared light. The light goes a few centimetres into the brain and measures the colour of the red blood cells as it changes with oxygenation. The oximeter does that every 5 seconds and the value of oxygenation is shown on the oximeter at all times.

***How will this change the treatment of my baby?***

If the oximeter shows a low value, the doctor will adjust the treatment and support that your baby receives. The adjustments follow a guideline that lists the ways treatment may be used to respond to a low oxygen in the brain. These treatments are all used in routine clinical practice in order to support respiration, blood circulation, and blood transport of gasses (oxygen, carbondioxide). The only addition now is that the use of treatments is guided also by the oxygen levels in the brain.

***How will the research be done?***

SafeBoosC-III is a randomised clinical trial. Many treatments are tested by randomised clinical trials, as it is the most reliable way to find out if they are effective.

A randomised clinical trial means that the decision about whether the baby will receive monitoring of brain oxygenation will be determined by chance, like the toss of a coin. This means that half of the babies that will participate in the SafeBoosC-III trial will receive treatment guided by monitoring of the brain oxygenation (experimental group) while the other half will receive treatment as usual, i.e. no monitoring of the brain (control group). We need 1600 babies from approximately 50 hospitals in Europe, USA and China to evaluate the intervention effect.

***Are there risks?***

Near-infrared light is difficult to see but can go some centimetres into the body. It is more like heat than light. The heat is less than that from the sun on a normal summer day and thus, it has no risks to the brain. There is, however, a small risk of skin burns, like on a sunny day. This risk can be minimised by moving the sensor at intervals. The nurse will do this very carefully in order not to disturb the child.

We hope that the specific guidelines (the adjustments to treatment) will prove beneficial to the baby, i.e. increase the chance of survival and reduce the risk of brain injury. When a baby is born too early, there is a risk that it may die or suffer damage to the brain, lungs, intestines or eyes. What we test in this trial is a new type of monitoring which is increasingly being used in neonatal departments, for instance in Holland, USA and Finland. General experience shows that new interventions may be better in some ways and yet can cause an increased risk in other ways. For the SafeBoosC-III trial it could be better for the brain but worse for the lungs or the eyes. We do not know at present. That is why it is so important to do randomised trials to clarify risks and benefits. So, as in all research, there may be unforeseen risks. All unexpected adverse events will be reported as a part of the research.

All observations, monitoring, and treatments will be as usual in the neonatal department. In particular, ultrasound scanning of the brain will be conducted several times as part of normal practice.

The investigators will access your baby’s hospital records for trial purposes, i.e. trial completion, surveillance and control of trial.

Any information that we collect from the hospital records will be kept confidential and in a secure place. A pseudoanonymised copy (identified only by a study number) will be sent to the trial centre in Copenhagen for statistics. Only authorised people involved in the trial will have access to data from your baby.

***Ancillary studies***

We are planning to roll out an ancillary study to follow up on your baby’s progress. Therefore, you may be contacted within the first two years of your baby’s life. Follow up may include a doctor’s examination, a questionnaire or a developmental test. You will be asked for separate consent for this or any other study that may be relevant for babies that take part in the SafeBoosC trial.

***Does my baby have to take part in SafeBoosC-III?***

No. It is your decision whether or not your baby should take part in the SafeBoosC-III trial. If you decide not to ‘opt-out’, you may change your mind later without having to give a reason and without it having any consequences to your baby's future care in any way.

If you decide that you do not want your baby to join the SafeBoosC-III trial at all, he or she will still be given the highest level of care and attention by staff.

***Exclusion from or termination of the trial***

If the cerebral oximetry monitoring causes unexpected problems for your baby, it will be stopped. With your permission, we will still want to include the data of your baby in the evaluation of the trial.

The trial is monitored by an external committee. If it appears that there are important unexpected problems or it appear that the trial will not be able to answer the questions that it was designed to answer, the trial may be stopped.

***Will I be told the results of SafeBoosC if we join?***

Yes. If you decide that you would like your baby to join SafeBoosC-III we will keep in touch with you to tell you the results of the trial when they become available (in year 2021-22), if you wish so.

The results from the SafeBoosC trial will, regardless of positive, negative, or inconclusive, be published in a public accessible journal.

***Funding for the trial***

The trial was planned by our group of European, academic neonatologists and funded by the Elsass Foundation of Denmark with a total amount of 0.4 mio euro to cover the central trial costs.

The trial sponsor Prof. Gorm Greisen or any other investigator in the trial, has no personal attachment to Elsass Foundation or any other organisation/company with interest in near-infrared spectroscopy monitoring.

Families will not be payed a fee for participation.

***The SafeBoosC-III trial has been authorised by (insert name of research ethics committee)***

***Thank you***

Thank you for taking the time to read this leaflet. If you would like more information about SafeBoosC-III, please ask your doctor or nurse.

**Contact Information the SafeBoosC-III trial**

**Sponsor:** Prof. Gorm Greisen

Address: Neonatalklinikken GN, Blegdamsvej 9, 2100 København Ø, Denmark

Telephone: +45 35 45 43 20

**Local contact person**

Address:

Telephone:

Prior informed consent – parental information for postnatal recruitment

*Information to parents who have given birth to a baby extremely preterm*

*Regarding the trial****:*** **SafeBoosC-III: Safeguarding the brain of our smallest children**

*An investigator-initiated, pragmatic, open label, multinational randomized phase III clinical trial evaluating treatment based on near-infrared spectroscopy monitoring versus treatment as usual in premature infants.*

***Dear Parent***

You are receiving this leaflet because your baby has been born very early and needs intensive care. We understand that this may be a stressful time for you and that it can be difficult to take in information at this time. This leaflet gives you information about a clinical trial called SafeBoosC-III. We are conducting this trial to investigate if monitoring of the brain’s oxygen levels can optimize intensive care treatment and thereby, improve the care of babies in the same situation as yours.

We ask for your permission to include your baby in the trial now because we need to start as soon as possible after birth. You are free to decide.

***What does trial participation involve?***

If your baby gets randomised to the control group, i.e. is randomly selected, he or she will receive the usual treatment and support given in the neonatal intensive care unit. This means he/she will still be given the highest level of care and attention by staff.

If, on the other hand, your baby gets randomised to the experimental group, he or she will also have a small sensor put on the head. This has to be done within six hours after birth. The sensor will be in place until 72 hours after birth. If the sensor shows that there may be too little oxygen in the brain, the doctor may adjust the support of the baby’s breathing and circulation to try to improve it.

There are no extra examinations done, but data from your baby’s hospital records will be used to calculate the result of the trial. The trial is multinational and has also been approved by an ethics review board in your country.

***What are we trying to find out?***

We want to know if monitoring of the brain’s oxygenation (keeping an eye on the amount of oxygen in the brain) can increase the chance of survival and decrease the risk of brain injury. The trial is based on results from a previous trial, SafeBoosC-II, where monitoring of brain oxygenation appeared to be beneficial. However, we need to test this on a larger scale. If it is successful, it may help prevent death or a life with handicap for at least 2,000 babies born much too early every year world-wide.

***This is the essential information. On the next pages you can read more details and read the standard conditions for participation in research projects. If you want to know more you can find the full protocol and other information about the trial on*** [***www.safeboosc.eu***](http://www.safeboosc.eu)***. Do not hesitate to ask questions.***

***In the following pages you can read more details about the SafeBoosC-III trial.***

***How can we know the oxygenation of the brain?***

We use an instrument called a near-infrared oximeter. It has a thin cable attached to a sensor (a small, soft patch). The sensor is put on the head of the baby and held in place by a bandage. The sensor uses near-infrared light. The light goes a few centimetres into the brain and measures the colour of the red blood cells as it changes with oxygenation. The oximeter does that every 5 seconds and the value of oxygenation is shown on the oximeter at all times.

***How will this change the treatment of my baby?***

If the oximeter shows a low value, the doctor will adjust the treatment and support that your baby receives. The adjustments follow a guideline that lists the ways treatment may be used to respond to a low oxygen in the brain. These treatments are all used in routine clinical practice in order to support respiration, blood circulation, and blood transport of gasses (oxygen, carbondioxide). The only addition now is that the use of treatments is guided also by the oxygen levels in the brain.

***How will the research be done?***

SafeBoosC-III is a randomised clinical trial. Many treatments are tested by randomised clinical trials, as it is the most reliable way to find out if they are effective.

A randomised clinical trial means that the decision about whether the baby will receive monitoring of brain oxygenation will be determined by chance, like the toss of a coin. This means that half of the babies participating in the SafeBoosC-III trial will receive treatment guided by monitoring of the brain oxygenation (experimental group) while the other half will receive treatment as usual, i.e. no monitoring of the brain (control group). We need 1600 babies from approximately 50 hospitals in Europe, USA and China to evaluate the intervention effect.

***Are there risks?***

Near-infrared light is difficult to see but can go some centimetres into the body. It is more like heat than light. The heat is less than that from the sun on a normal summer day and thus, it has no risks to the brain. There is, however, a small risk of skin burns, like on a sunny day. In the previous trial, SafeBoosC-II skin marks were seen in 10% of babies. In very rare cases this may lead to a scar. This risk can be minimised by moving the sensor at intervals. The nurse will do this very carefully in order not to disturb your baby.

We do the trial because with think that the specific guidelines (the adjustments to treatment) will prove beneficial to the baby, i.e. increase the chance of survival and reduce the risk of brain injury. It is already in routine use in neonatal departments, for instance in Holland, the US and Finland. When a baby is born too early, however, there is a risk that he/she may die or suffer damage to the brain, lungs, intestines or eyes. General experience shows that new interventions may be better in some ways and yet can cause an increased risk in other ways. For the SafeBoosC-III trial it could reduce the risk of death and be better for the brain but worse for the lungs or the eyes. We do not know at present. That is why it is necessary to test it properly in a randomised trial to clarify risks and benefits before taking it into routine use. So, as in all research, there may be unforeseen risks. All severe adverse reactions will be reported as a part of the research to make sure that we learn as much as possible from it.

All observations, treatments and additional monitoring will be as usual in the neonatal department.

The investigators will access your baby’s hospital records for trial purposes, i.e. trial completion, surveillance and control of trial.

Any information that we collect from the hospital records will be kept confidential and in a secure place. A pseudoanonymised copy (identified only by a study number, so your child cannot be identified) will be sent to the trial centre in Copenhagen for statistics. Only authorised people involved in the trial will have access to data from your baby.

***Follow-up studies***

We are planning to roll out an additional study to follow up on your baby’s progress. Therefore, you may be contacted within the first two years of your baby’s life. Follow up may include a doctor’s examination, a questionnaire or a developmental test. You will be asked for separate consent for this or any other study that may be relevant for babies that take part in the SafeBoosC trial.

***Does my baby have to take part in SafeBoosC-III?***

No. It is your decision whether or not your baby should take part in the SafeBoosC-III trial. If you decide to enrol your baby in the trial, you may change your mind later without having to give a reason and without it having any consequences to your baby's future care in any way.

If you decide that you do not want your baby to join the SafeBoosC-III trial at all, he or she will still be given the highest level of care and attention by staff.

***Exclusion from or termination of the trial***

If the cerebral oximetry monitoring causes unexpected problems for your baby, it will be stopped. With your permission, we will still want to include the data of your baby in the evaluation of the trial.

The trial is monitored by an external committee. If it appears that there are important unexpected problems or it appear that the trial will not be able to answer the questions that it was designed to answer, the trial may be stopped.

***Will I be told the results of SafeBoosC if we join?***

Yes. If you decide that you would like your baby to join SafeBoosC-III we will keep in touch with you to tell you the results of the trial when they become available (in year 2021-22), if you wish so.

The results from the SafeBoosC trial will, regardless of positive, negative, or inconclusive, be published in a public accessible journal.

***What will happen to the data you have collected regarding my baby?***

The persons involved in this trial have no economic interest in any aspects of the trial. This trial is solely made to improve the care of babies born too early. Therefore, in order to optimize the value of your baby’s participation, we will store your baby’s anonymized data for at least 10 years so that other researchers may use them to examine other research questions in the same field.

***Funding for the trial***

The trial was planned by our group of European, academic neonatologists and funded by the Elsass Foundation of Denmark with a total amount of 0.4 mio euro to cover the central trial costs. We are applying for further funding at the Medtronics Foundation and the Novo Nordisk Foundation.

The trial sponsor Prof. Gorm Greisen or any other investigator in the trial, has no personal attachment to Elsass Foundation, Medtronics Foundation, Novo Nordisk Foundation or any other organisation/company with interest in near-infrared spectroscopy monitoring.

Families will not be payed a fee for participation.

***The SafeBoosC-III trial has been authorised by (insert name of research ethics committee)***

***Thank you***

Thank you for taking the time to read this leaflet. If you would like more information about SafeBoosC-III, please ask your doctor or nurse.

**Contact Information the SafeBoosC-III trial**

**Sponsor:** Prof. Gorm Greisen

Address: Neonatalklinikken GN, Blegdamsvej 9, 2100 København Ø, Denmark

Telephone: +45 35 45 43 20

**Local contact person**

Address:

Telephone:

Prior informed consent – parental information for prenatal recruitment

*Information to parents who may give birth to a baby extremely preterm*

*Regarding the trial****:*** **SafeBoosC-III: Safeguarding the brain of our smallest children**

*An investigator-initiated, pragmatic, open label, multinational randomized phase III clinical trial evaluating treatment based on near-infrared spectroscopy monitoring versus treatment as usual in premature infants.*

***Dear Parent***

You are receiving this leaflet because your baby may be born very early and may need intensive care. We understand that this may be a stressful time for you and that it can be difficult to take in information at this time. This leaflet gives you information about a clinical trial called SafeBoosC-III. We are conducting this trial to investigate if monitoring of the brain’s oxygen levels can optimize intensive care treatment and thereby, improve the care of babies in the same situation as yours.

We ask for your permission to include your baby in the trial now because we need to start as soon as possible after birth. You are free to decide.

***What does trial participation involve?***

If your baby gets randomised to the control group, i.e. is randomly selected, he or she will receive the usual treatment and support given in the neonatal intensive care unit. This means he/she will still be given the highest level of care and attention by staff.

If, on the other hand, your baby gets randomised to the experimental group, he or she will also have a small sensor put on the head. This has to be done within six hours after birth. The sensor will be in place until 72 hours after birth. If the sensor shows that there may be too little oxygen in the brain, the doctor may adjust the support of the baby’s breathing and circulation to try to improve it.

There are no extra examinations done, but data from your baby’s hospital records will be used to calculate the result of the trial. The trial is multinational and has also been approved by an ethics review board in your country.

***What are we trying to find out?***

We want to know if monitoring of the brain’s oxygenation (keeping an eye on the amount of oxygen in the brain) can increase the chance of survival and decrease the risk of brain injury. The trial is based on results from a previous trial, SafeBoosC-II, where monitoring of brain oxygenation appeared to be beneficial. However, we need to test this on a larger scale. If it is successful, it may help prevent death or a life with handicap for at least 2,000 babies born much too early every year world-wide.

***This is the essential information. On the next pages you can read more details and read the standard conditions for participation in research projects. If you want to know more you can find the full protocol and other information about the trial on*** [***www.safeboosc.eu***](http://www.safeboosc.eu)***. Do not hesitate to ask questions.***

***In the following pages you can read more details about the SafeBoosC-III trial.***

***How can we know the oxygenation of the brain?***

We use an instrument called a near-infrared oximeter. It has a thin cable attached to a sensor (a small, soft patch). The sensor is put on the head of the baby and held in place by a bandage. The sensor uses near-infrared light. The light goes a few centimetres into the brain and measures the colour of the red blood cells as it changes with oxygenation. The oximeter does that every 5 seconds and the value of oxygenation is shown on the oximeter at all times.

***How will this change the treatment of my baby?***

If the oximeter shows a low value, the doctor will adjust the treatment and support that your baby receives. The adjustments follow a guideline that lists the ways treatment may be used to respond to a low oxygen in the brain. These treatments are all used in routine clinical practice in order to support respiration, blood circulation, and blood transport of gasses (oxygen, carbondioxide). The only addition now is that the use of treatments is guided also by the oxygen levels in the brain.

***How will the research be done?***

SafeBoosC-III is a randomised clinical trial. Many treatments are tested by randomised clinical trials, as it is the most reliable way to find out if they are effective.

A randomised clinical trial means that the decision about whether the baby will receive monitoring of brain oxygenation will be determined by chance, like the toss of a coin. This means that half of the babies participating in the SafeBoosC-III trial will receive treatment guided by monitoring of the brain oxygenation (experimental group) while the other half will receive treatment as usual, i.e. no monitoring of the brain (control group). We need 1600 babies from approximately 50 hospitals in Europe, USA and China to evaluate the intervention effect.

***Are there risks?***

Near-infrared light is difficult to see but can go some centimetres into the body. It is more like heat than light. The heat is less than that from the sun on a normal summer day and thus, it has no risks to the brain. There is, however, a small risk of skin burns, like on a sunny day. In the previous trial, SafeBoosC-II skin marks were seen in 10% of babies. In very rare cases this may lead to a scar. This risk can be minimised by moving the sensor at intervals. The nurse will do this very carefully in order not to disturb your baby.

We do the trial because with think that the specific guidelines (the adjustments to treatment) will prove beneficial to the baby, i.e. increase the chance of survival and reduce the risk of brain injury. It is already in routine use in neonatal departments, for instance in Holland, the US and Finland. When a baby is born too early, however, there is a risk that he/she may die or suffer damage to the brain, lungs, intestines or eyes. General experience shows that new interventions may be better in some ways and yet can cause an increased risk in other ways. For the SafeBoosC-III trial it could reduce the risk of death and be better for the brain but worse for the lungs or the eyes. We do not know at present. That is why it is necessary to test it properly in a randomised trial to clarify risks and benefits before taking it into routine use. So, as in all research, there may be unforeseen risks. All severe adverse reactions will be reported as a part of the research to make sure that we learn as much as possible from it.

All observations, treatments and additional monitoring will be as usual in the neonatal department.

The investigators will access your baby’s hospital records for trial purposes, i.e. trial completion, surveillance and control of trial.

Any information that we collect from the hospital records will be kept confidential and in a secure place. A pseudoanonymised copy (identified only by a study number, so your child cannot be identified) will be sent to the trial centre in Copenhagen for statistics. Only authorised people involved in the trial will have access to data from your baby.

***Follow-up studies***

We are planning to roll out an additional study to follow up on your baby’s progress. Therefore, you may be contacted within the first two years of your baby’s life. Follow up may include a doctor’s examination, a questionnaire or a developmental test. You will be asked for separate consent for this or any other study that may be relevant for babies that take part in the SafeBoosC trial.

***Does my baby have to take part in SafeBoosC-III?***

No. It is your decision whether or not your baby should take part in the SafeBoosC-III trial. If you decide to enrol your baby in the trial, you may change your mind later without having to give a reason and without it having any consequences to your baby's future care in any way.

If you decide that you do not want your baby to join the SafeBoosC-III trial at all, he or she will still be given the highest level of care and attention by staff.

***Exclusion from or termination of the trial***

If the cerebral oximetry monitoring causes unexpected problems for your baby, it will be stopped. With your permission, we will still want to include the data of your baby in the evaluation of the trial.

The trial is monitored by an external committee. If it appears that there are important unexpected problems or it appear that the trial will not be able to answer the questions that it was designed to answer, the trial may be stopped.

***Will I be told the results of SafeBoosC if we join?***

Yes. If you decide that you would like your baby to join SafeBoosC-III we will keep in touch with you to tell you the results of the trial when they become available (in year 2021-22), if you wish so.

The results from the SafeBoosC trial will, regardless of positive, negative, or inconclusive, be published in a public accessible journal.

***What will happen to the data you have collected regarding my baby?***

The persons involved in this trial have no economic interest in any aspects of the trial. This trial is solely made to improve the care of babies born too early. Therefore, in order to optimize the value of your baby’s participation, we will store your baby’s anonymized data for at least 10 years so that other researchers may use them to examine other research questions in the same field.

***Funding for the trial***

The trial was planned by our group of European, academic neonatologists and funded by the Elsass Foundation of Denmark with a total amount of 0.4 mio euro to cover the central trial costs. We are applying for further funding at the Medtronics Foundation and the Novo Nordisk Foundation.

The trial sponsor Prof. Gorm Greisen or any other investigator in the trial, has no personal attachment to Elsass Foundation, Medtronics Foundation, Novo Nordisk Foundation or any other organisation/company with interest in near-infrared spectroscopy monitoring.

Families will not be payed a fee for participation.

***The SafeBoosC-III trial has been authorised by (insert name of research ethics committee)***

***Thank you***

Thank you for taking the time to read this leaflet. If you would like more information about SafeBoosC-III, please ask your doctor or nurse.

**Contact Information the SafeBoosC-III trial**

**Sponsor:** Prof. Gorm Greisen

Address: Neonatalklinikken GN, Blegdamsvej 9, 2100 København Ø, Denmark

Telephone: +45 35 45 43 20

**Local contact person**

Address:

Telephone:

**Informed consent – the SafeBoosC-III trial**

**(To be adapted for each country submission to the regulatory authorities)**

***Title of the trial:***

SafeBoosC-III: Safeguarding the brain of our smallest children.

*An investigator-initiated, pragmatic, open label, multinational randomized phase III clinical trial evaluating treatment based on near-infrared spectroscopy monitoring versus treatment as usual in premature infants.*

***Informed consent***

I/we have been fully informed by the attending doctor (investigator) of the potential favorable and adverse effects of the trial. I/we understand the aims, randomisation procedure, intervention, significance and relevance of the SafeBoosC-III trial. The trial is in the interests of the smallest of our children’s health.

I/we have been informed that participation is voluntary and that I/we can withdraw my/our consent at any time without having to provide reasons and without incurring any disadvantages for my/our child or myself/us as a result.

I/we have been informed that insurance for participants has been taken care of. This is intended to cover any unforeseen possible injuries that could arise from the participation of my/our child in the trial.

I am/we are aware that by signing this paper of informed consent, we are allowing investigators and the external monitoring personnel to access our baby’s hospital records and transfer of data from the records to the Copenhagen Trial Unit in Denmark for statistical calculations, as described in the parental information leaflet.

I/we have read and understood the text of the parental information and the informed consent. At the time of the information consultation my/our questions were answered clearly and adequately by the investigator (or delegated personnel). I/we confirm with my/ours signature(s) that I/we am/are willing to let my/our child participate in the SafeBoosC-III trial. I/we have received a copy of this declaration of consent (signed) and the accompanying parental information.

**Participant / Child ID:.........................................**

**Parent (s)**

Both parents’ signatures are required

Name:.................................................................Signature:..............................................................

Place/Date:.................................................................

Name:.................................................................Signature:..............................................................

Place/Date:.................................................................

**National coordinating Investigator (or delegated personnel) providing the parental information**

I hereby confirm that the parent/parents to the participant have been given both verbal and written information about the trial, have been given as much time as possible, given the urgency of trial intervention, to consider the child’s participation, and, have been given the opportunity to ask questions about the participation. Furthermore, I am convinced the parent/parents have been given sufficient information and that the information is understood, such as informed consent can be given.

Name:.................................................................Signature:..............................................................

Place/Date:.................................................................

Sponsor of the SafeBoosC study:

Professor. Gorm Greisen

Rigshopitalet Copenhagen, Dept. Neonatology

Blegdamsvej 3,

DK-2100 Copenhagen, Denmark
