# Supplementary material for: Cerebral near-infrared spectroscopy monitoring versus treatment as usual for extremely preterm infants: a protocol for the SafeBoosC randomised clinical phase III trial
Source: Trials. 2019 Dec 30;20:811. doi: 10.1186/s13063-019-3955-6 (PMC6937938; doi:10.1186/s13063-019-3955-6)
Supplement: Supplementary file 4 — Additional file 4. WHO trial registration data set. [file 13063_2019_3955_MOESM4_ESM.docx]

**World Health Organization Trial Registration Data Set**

| Primary Registry and Trial Identifying Number | clinicaltrials.gov (NCT03770741) |
| --- | --- |
| Date of Registration in Primary Registry | 07/12-2018 |
| Secondary Identifying Numbers | N/A |
| Source(s) of Monetary or Material Support | Elsass Foundation (2,7 mill DKK)  Svend Andersen Foundation (1,0 mill DKK)  Aage og Johanne-Louis Hansen Foundation (1,0 mill DKK) |
| Primary Sponsor | Region Hovedstaden, Department of Neonatology 5024, Rigshospitalet, Blegdamsvej 9, 2100 Copenhagen Ø, Denmark. |
| Secondary Sponsor(s) | N/A |
| Contact for Public Queries | Phone +45 3545 1326, e-mail: [gorm.greisen@regionh.dk](mailto:gorm.greisen@regionh.dk) |
| Contact for Scientific Queries | Professor Gorm Greisen, Department of Neonatology, Rigshospitalet, Blegdamsvej 9, 2100 Copenhagen Ø, Denmark. Phone: +45 3545 1326, e-mail: [gorm.greisen@regionh.dk](mailto:gorm.greisen@regionh.dk) |
| Public Title | Safeguarding the brain of our smallest children |
| Scientific Title | SafeBoosC-III - Safeguarding the brain of our smallest children – an investigator-initiated, pragmatic, open label, multinational randomized phase III clinical trial evaluating treatment based on near-infrared spectroscopy monitoring versus  treatment as usual in premature infants |
| Countries of Recruitment | Austria, Belgium, China, Czech Republic, Denmark, England, France, Germany, Greece, India, Ireland, Italy, Norway, Poland, Portugal, Switzerland, Scotland, Spain, Turkey, USA |
| Health Condition(s) or Problem(s) Studied | Death and brain injury |
| Intervention(s) | Two arms   - Experimental group/NIRS group: participants in the experimental group will be monitored during the first 72 hours of life with a cerebral NIRS oximeter. Cerebral hypoxia will be treated according to an evidence-based treatment guideline - Control group: participants in the control group will not undergo cerebral oxygenation monitoring and will receive treatment as usual |
| **Key Inclusion and Exclusion Criteria** | **Inclusion criteria**  The inclusion criteria are: infants born before 28 weeks postmenstrual age and signed parental informed consent, unless the NICU has chosen to use ‘opt-out’ or deferred consent as their consent method.  **Exclusion criteria**  The exclusion criteria are: missing signed parental informed consent (or if the ‘opt-out’ method is used, lack of a record that the clinical staff have explained the trial and the ‘opt-out’ consent process to parents and/or a record in the infant’s clinical file of parents’ decision to opt-out); decision not to provide full life support; and no possibility to initiate cerebral NIRS monitoring within six hours after birth. |
| **Study Type** | Investigator-initiated multinational randomized, pragmatic phase III clinical trial with a two-parallel group design. It is an open label trial, but parts will be conducted blinded to intervention (see protocol and SPIRIT checklist).  The objective of this trial is to examine the benefits and harms of treatment based on NIRS monitoring compared with treatment as usual (standard monitoring and treatment) to reduce cerebral hypoxia during the first 72 hours of life in extremely preterm infants. The hypothesis is that the application of treatment based on NIRS monitoring will decrease a composite outcome of severe brain injury or death at 36 weeks postmenstrual age. |
| **Date of First Enrollment** | **June 2019** |
| **Sample Size** | **We will enroll 1600 participants** |
| **Recruitment Status** | **Active** |
| **Primary Outcome(s)** | **Outcome name: death or severe brain injury**  **Metric/method of measurement: death as registered in clinical file up until follow-up, severe brain injury as any of the following diagnoses on cranial ultrasound;** cerebral intraventricular haemorrhage grade III or IV, cystic periventricular leukomalacia, cerebellar haemorrhage, post-haemorrhagic ventricular dilatation, or cerebral atrophy up until follow-up.  Time point: 36 weeks of postmenstrual gestational age |
| **Secondary outcome(s)** | **None** |
| **Exploratory outcome(s)** | **Exploratory outcome no 1**   - **Outcome name: major neonatal morbidities count** - **Metric/method of measurement:** a score of the presence of bronchopulmonary dysplasia, retinopathy of prematurity stage 3+, and severe brain injury as defined in the primary outcome, registered in clinical file up until follow-up - **Time point: 36 weeks postmenstrual age**   **Exploratory outcome no 2**   - **Outcome name:** bronchopulmonary dysplasia - **Metric/method of measurement:** oxygen or ventilator/continuous positive airway pressure (CPAP) requirement, registered in clinical file up until time of assessment. - Time point: 36 weeks postmenstrual age   Exploratory outcome no 3   - **Outcome name:** retinopathy of prematurity - **Metric/method of measurement: stage 3 or higher registered in clinical file up until time of assessment** - **Time point: 36 weeks postmenstrual age**   **Exploratory outcome no 4**   - Outcome name: necrotising enterocolitis - Metric/method of measurement: Score of 2 or higher using the modified Bell’s staging and/or focal intestinal perforation registered in clinical file, up until time of assessment - Time point: 36 weeks postmenstrual age   **Exploratory outcome no 5**   - **Outcome name: late-onset sepsis** - **Metric/method of measurement: being treated with antibiotics a minimum of five days registered in clinical file, up until time of assessment** - **Time point: 36 weeks postmenstrual age** |
| **Ethics Review** | **Approved in 57 hospitals** |
| **Completion date** | **N/A** |
| **Summary Results** | **N/A** |
| **IPD sharing statement** | **Undecided** |
